# Diffusion-Induced Stress in Commercial Graphite Electrodes during Multiple Cycles Measured by an In Situ Method

**DOI:** 10.3390/mi13010142

**Published:** 2022-01-17

**Authors:** Dawei Li, Guanglin Zhu, Huibing Liu, Yikai Wang

**Affiliations:** 1 School of Mechanical Engineering, University of Shanghai for Science and Technology, Shanghai 200093, China; zgl3913@126.com; 2 Shanghai Institute of Applied Mathematics and Mechanics, School of Mechanics and Engineering Science, Shanghai University, Shanghai 200444, China; huibingliu@shu.edu.cn; 3 Department of Chemical and Materials Engineering, University of Kentucky, Lexington, KY 40506, USA

**Keywords:** stress evolution, modulus variation, composite electrode, in situ measurement, lithium batteries

## Abstract

The cyclic stress evolution induced by repeated volume variation causes mechanical degradation and damage to electrodes, resulting in reduced performance and lifetime of LIBs. To probe the electro-chemo-mechanical coupled degradation, we conducted in situ measurements of Young’s modulus and stress evolution of commercial used graphite electrodes during multiple cycles. A bilayer graphite electrode cantilever is cycled galvanostatically in a custom cell, while the bending deformation of the bilayer electrode is captured by a CCD optical system. Combined with a mechanical model, Li-concentration-dependent elastic modulus and stress are derived from the curvature of the cantilever electrode. The results show that modulus, stress and strain all increase with the lithium concentration, and the stress transforms from compression to tension in the thickness direction. During multiple cycles, the modulus decreases with an increase in the cycle number at the same concentration. The maximum stress/strain of each cycle is maintained at almost same level, exhibiting a threshold that results from the co-interaction of concentration and damage. These findings provide basic information for modeling the degradation of LIBs.

## 1. Introduction

Lithium-ion batteries (LIBs) are primary power sources for portable electronic devices and electric vehicles. Nevertheless, the energy density and durability of current LIBs are unable to satisfy the increasingly heightened expectations and the rapidly developing electronic industry [1,2,3,4]. Mechanical properties and stress are crucial to the performance and lifespan of LIBs. For further commercialization of the battery materials with high energy density, such as Si and Li metal, the severe mechanical degradation during cycling should be in depth understood [5,6]. Insertion and extraction of Li ions within the active materials could induce cyclic volumetric expansion and contraction, giving rise to stress and damage in electrodes, which lead to capacity fade and declination of lifetime [7,8,9]. Therefore, unveiling the role and impact of the mechanical properties and stress in the degradation and performance of electrodes is indispensable to optimize the performance of LIBs.

Several works have studied the mechanical response of battery electrodes during electrochemical cycling. The results have help get in depth understanding of the microstructure, composition and surface morphology of electrodes. Multiple techniques were employed, such as: TEM, SEM, AFM, XTM, etc. [10,11,12,13,14,15,16,17]. Liu et al. [10] studied the volume expansion of Si nanowires, SnO_2_ nanowires and Si nanoparticles using TEM. The results revealed that the active materials could avoid facture when the diameter of particle is below 150 nm. Dahn et al. [14] studied the deformation and cracking of lithium thin films by combining optical microscopy and AFM. They found that the cracks in the thin film are mainly caused by lateral contraction during delithiation. Additionally, the XTM was used to detect the working mechanism of Sn based electrodes. The results showed that Sn particles would form a core–shell internal structure during lithiation/delithiation cycling.

Furthermore, in situ measurement techniques were developed to investigate the mechanical degradation stemming from the stress evolution. There are three main methods for in situ measurements of strain and stress, including digital image correlation (DIC) [18,19,20], multiple-optical-stress sensor (MOSS) [21,22,23,24,25,26,27] and curvature measurement system (CMS) [28,29,30]. Qi and Harris [18] implied that the volume expansion of graphite particles during lithium intercalation is mainly accommodated by a decrease in porosity employing DIC. Jones et al. improved DIC by laser and speckle pattern to investigate the strain variation of a free-standing composite graphite electrode in real time [19]. DIC can map full-field strains of a whole electrode but is unable to measure stress directly. MOSS is widely used to explore thin film electrodes in lithium batteries. Sethuraman et al. revealed the variation of compressive and tensile stress, and the relation between stress change and potential change, and finding that the modulus decreases with degree of lithiation [21,22,23]. Besides, MOSS was also used to investigate the development of irreversible compressive stresses in graphitic carbon thin film electrodes. Mukhopadhyay et al. found that SEI layer formation rather than Li ion intercalation is the primary reason for irreversible stress [25,26,27]. MOSS can observe stress evolution of a thin film electrode but cannot be widely applied to analyze the working mechanism of commercial composite electrodes. Recently, Li et al. conducted in situ measurements of composite graphite electrodes by curvature measurement system, establishing the relationship among the deformation, modulus, partial molar volume and state of charge [29]. This system is suitable for analyzing the working mechanism of commercial used electrodes in a custom cell.

Complementing the experimental studies, numerical models have been developed to model Li ion diffusion-induced deformation and stress during cycling. Cheng and Verbrugge [31] formulated analytical equations for the evolution of stress and strain in a spherical particle under both galvanostatic and potentiostatic conditions. Bower et al. [32] developed a finite strain model governing the coupled processes of diffusion, stress, deformation, plastic flow, and electrochemical reactions in a Li-ion half-cell, finding that the stress directly influences the potential at the interface. Bhandakkar and Gao [33] presented a cohesive model in an initially crack-free strip electrode under galvanostatic cycling, and identified a critical characteristic dimension of crack nucleation. These models are helpful to understand the chemo-mechanical coupled degradation of electrode at material level. However, it is difficult to predict the stress and mechanical properties via theoretical model, due to commercial composite electrodes featured with heterogeneous micropores.

In this paper, we developed the curvature measurement system to get in situ measurements of the bending deformation, elastic modulus, stress and strain evolution of composite graphite electrodes during multiple cycles. The deformation as a function of Li-ion concentration during galvanostatic cycling is monitored in real time by cantilever-based custom battery cell with charge-coupled device (CCD) optical acquisition system. A mechanical model is proposed to quantify the evolutions of the elastic modulus, stress and strain. Conducting multiple-cycle analysis provides more insight into electro-chemo-mechanical coupled degradation mechanism associated with the stress development.

## 2. Experiment

### 2.1. Electrochemical Cell Assembly

**Figure 1:**
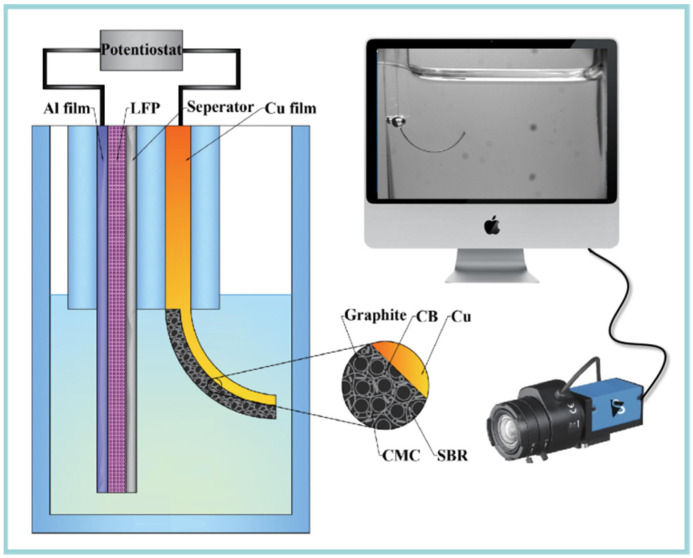
Schematic of the in situ measurement system, which consist an electrochemical cell, CCD camera and a computer.

Combining an in situ observation system, a bilayer cantilever was designed to get the relationship between curvature (κ) and state of charge (SOC) during electrochemical cycling. Here, a transparent quartz cell was designed to get in situ capture of the bending deformation of the bilayer electrode with the help of a CCD video camera, as shown in Figure 1. Commercial used graphite electrode (Shenzhen Kejing Star Technology Company Ltd., Shenzhen, China) was used as the working electrode, which was made by mixing carbonaceous mesophase spherules graphite, styrene-butadiene rubber (SBR), carboxymethyl cellulose (CMC) and carbon black (CB) with a weight ratio of 94.5:2.25:2.25:1. The theoretical capacity and thickness are 330 mAh/g and 94 µm, respectively. A LiFePO_4_ (LFP) electrode was used as the positive electrode.

To build a bilayer cantilever, the graphite electrode was cut into 30 mm × 3 mm stripes, while the LFP electrode was made in a trios of 60 mm × 6 mm, whose capacity was large enough to supply enough lithium ions. A woven Celgard 2400 separator with a thickness of 25 μm was used to avoid the short circuit. Then, the two types of electrodes were fully immersed in the electrolyte, with 1 M LiPF6 in a mixture of ethylene carbonate and diethyl carbonate with 1:1 volume (Kejing Star Technology Company Ltd., Shenzhen, China). All the assemble procedures were performed inside an argon-filled glovebox.

### 2.2. Electrochemical Test

The cell was rested in the glovebox for 12 h, and was then cycled to analyze the working mechanism of commercial graphite electrode by a BKB6808 battery test system. The electrochemical cycles were conducted in a galvanostatical made with a constant current of 356 μA (ca. C/10 C-rate). The charging time was set as 4 h to reach a normalized capacity of 40%. The cut-off voltage was set as 4.2 V for charging process and 2.0 V for discharging process, respectively. Hence, the charging/discharging process would end when either the prescribed time or the prescribed cut-off voltage was attained. Meanwhile, the experiment data (voltage, current, and capacity) were recorded with a rate of 1 Hz during electrochemical cycles, simultaneously. The bending image was captured by a CCD camera every 1 min.

### 2.3. Model and Basic Equations of Curvature Change

**Figure 2:**
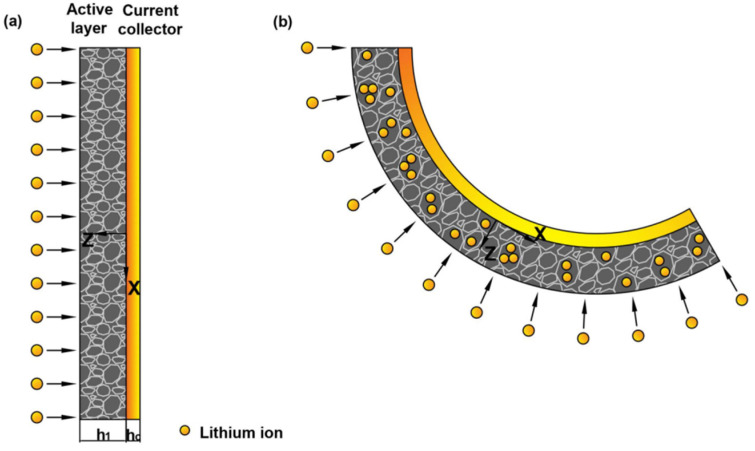
Schematic illustration of a bilayer cantilever electrode: (**a**) the pristine state of bilayer electrode, (**b**) deformed state with the insertion of Li ions.

Detailed working mechanisms between the electrochemical cycling and bending deformation can be provided by mechanical analysis [28]. Based on the deformation process in the experiment, we employed the geometry model in Figure 2. Here, $h_{1}$ and $h_{c}$ are the thickness of the active layer and current collector, respectively. During the electrochemical reaction, the insertion of Li ions can cause the expansion of graphite particle, while the extraction can cause shrinkage, respectively. Meanwhile, the current collector would restrict the volumetric change of the active layer. The mismatch could cause bending of the graphite electrode, as shown in Figure 2.

Usually, the commercial graphite electrodes in batteries are made from a mixture of active particles, conductive additive, binder and pores. All the constituents distribute randomly in the active layer, thus the localized deformation of the composite electrodes is relatively complex [34]. For simplification, the active layer is assumed to be macroscopically elastic, homogeneous and isotropic [35]. During the electrochemical cycles, the electrode is cycled with a relatively small current density. So the lithium concentration, c, can be assumed to be constant aligned with *z*-axis. According to the small deformation theory, the in-plain strain $\varepsilon_{0}$ and bending curvature κ of the electrode are [36],

(1) $$\begin{array}{l} \varepsilon_{0}=\frac{\Omega c\left(h_{1}^{4}E_{{}_{1}}^{}(c)+3h_{c}^{2}h_{1}^{2}E_{c}^{}+4h_{c}^{3}h_{1}E_{c}^{}\right)E_{1}(c)}{3\left(h_{1}^{4}E_{1}^{2}(c)+4h_{c}^{}h_{1}^{3}E_{c}^{}E_{1}(c)+6h_{c}^{2}h_{1}^{2}E_{c}^{}E_{1}(c)+4h_{c}^{3}h_{1}E_{c}^{}E_{1}(c)+h_{c}^{4}E_{c}^{2}\right)} \end{array}$$

(2) $$\begin{array}{l} \kappa =\frac{2\Omega c\left(h_{c}^{}h_{1}^{2}+h_{c}^{2}h_{1}^{}\right)E_{c}^{}E_{1}(c)}{\left(h_{1}^{4}E_{1}^{2}(c)+4h_{c}^{}h_{1}^{3}E_{c}^{}E_{1}(c)+6h_{c}^{2}h_{1}^{2}E_{c}^{}E_{1}(c)+4h_{c}^{3}h_{1}^{}E_{c}^{}E_{1}(c)+h_{c}^{4}E_{c}^{2}\right)} \end{array}$$

where, $E_{1}(c)$ and $E_{c}$ are the modulus of the active layer and the current collector, respectively. *Ω* is the partial molar volume of the active material. Resolve Equation (2), the modulus during the charging/discharging process can be expressed as:

(3) $$E_{1}(c)=\frac{E_{c}\kappa h_{c}{}^{4}}{\begin{array}{l} -(2\kappa h_{1}{}^{3}h_{c}+3\kappa h_{1}{}^{2}h_{c}{}^{2}+2\kappa h_{1}h_{c}{}^{3}-\Omega ch_{1}{}^{2}h_{c}-\Omega ch_{1}h_{c}{}^{2})\\ +\sqrt{{\left(2\kappa h_{1}{}^{3}h_{c}+3\kappa h_{1}{}^{2}h_{c}{}^{2}+2\kappa h_{1}h_{c}{}^{3}-\Omega ch_{1}{}^{2}h_{c}-\Omega ch_{1}h_{c}{}^{2}\right)}^{2}-\kappa^{2}h_{1}{}^{4}h_{c}{}^{4}} \end{array}}$$

Here, $E_{1}(c)$ is solution of the quadratic Equation (2), which indicates that the electrode curvature is related to material property parameters during cycling. Combining the curvature detected by video camera during cycles, with the thickness and volume expansion, the in situ evolution of modulus is monitored.

Then, the stress of the active layer $\sigma_{1}$ and the current collector $\sigma_{c}$ are calculated, respectively through the following constitutive equations,

(4) $$\begin{array}{l} \sigma_{1}=E_{1}(c)\left(\varepsilon_{0}+z\kappa \right)-\frac{1}{3}E_{1}(c)\Omega c\\ \sigma_{c}=E_{c}\left(\varepsilon_{0}+z\kappa \right) \end{array}$$

## 3. Results and Discussion

**Figure 3:**
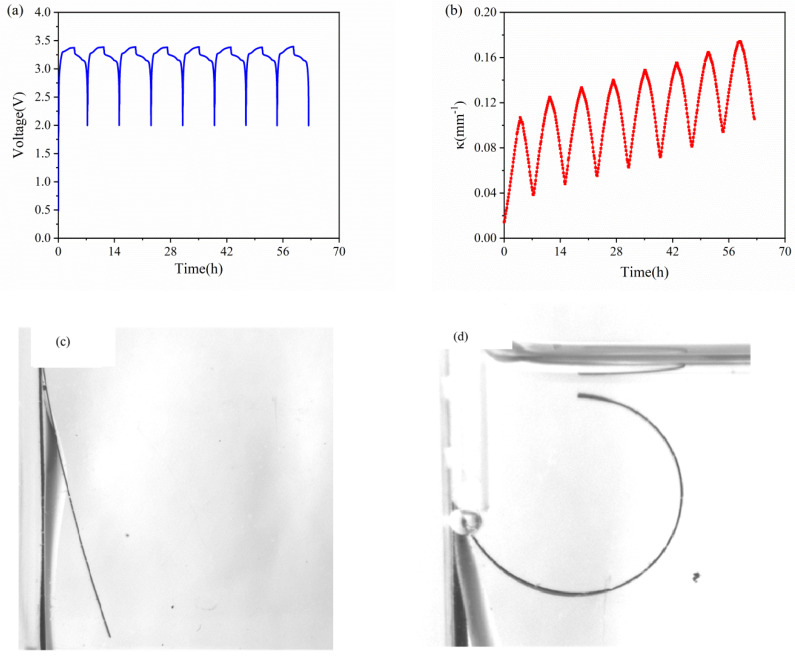
Voltage (**a**) and curvature (**b**) evolution of commercial graphite electrodes in the first 8 cycles. (**c**) pristine state and (**d**) bending deformation of bilayer elecrode after 5 cycles.

Figure 3a,b show the voltage and bending curvature of the composite graphite electrode against time in eight cycles. As the electrochemical cycles go on, the active plate undergoes periodical expansion and contraction. Meanwhile, it is restricted by the current collector, which can lead to periodical bending deformation. As shown in Figure 3b, the bending deformation increases in the charging process, and reverses in the discharging process. Along with the cycles, the curvature increases steadily. After each cycle, the bilayer electrode has small residual curvature. It is primarily associated with SEI formation and residual Li ions in graphite, as well as irreversible microstructure change and damage.

**Figure 4:**
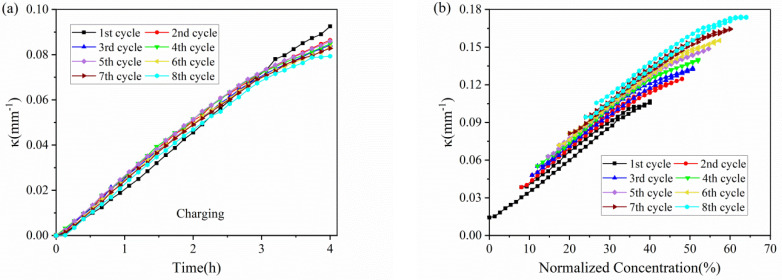
Curvature evolution of composite graphite electrodes in 8 cycles: (**a**) charging process with a zero initial bending curvature; (**b**) full cycle.

The evolution of the curvature in each cycle against time is shown in Figure 4. The results shown that the composite graphite electrodes have almost the same evolution trend in the charging process, thus the electrodes will endure a steady bending deformation without formation of cracks. During the charging process of each cycle, the curvature variation shows almost two stages: 1. it increases linearly in the first three hours; 2. its slope decrease in the last one hour. The discharging process shows the opposite trend. From the macro perspective, such phenomenon is attributed to the growing elastic modulus of the active materials. The modulus of graphite shows an increasing trend under continuous insertion of Li ions [28]. From micro perspective, as increasing Li ions insert into active particles, the pores for Li^+^ shuttling become further taken up and embedding paths become more winding as well, which block and mitigate the further lithiation and bending deformation. Additionally, it is found that the evolution rate as well as maximum curvature increment is almost same between the 2nd cycle and 8th cycle in both charging and discharging process, indicating the graphite electrode maintains excellent stability during electrochemical cycles.

The bending deformation can also be visualized by actual curvature with normalized concentration of lithium ions, as shown in Figure 4b. At the end of each cycle, the lithium ions cannot extract from the active particles, this will induce a residual curvature in the star of the cycle. However, the change in curvature and slope is consistent with that in Figure 4a, where we obtain the relationship of concentration, deformation and modulus. Moreover, the charging and discharging process exhibit parallel curvature evolution and multiple cycles keep in parallel with each other.

**Figure 5:**
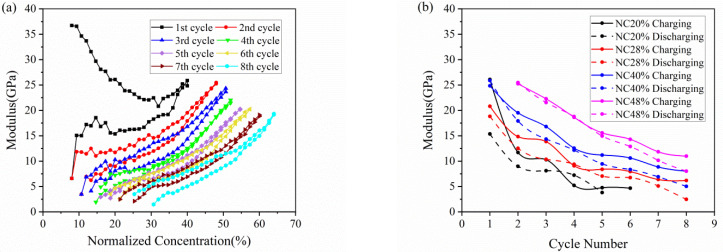
Modulus evolution of composite graphite electrodes corresponding to (**a**) normalized concentration and (**b**) cycle number in 8 cycles.

The elastic modulus of the active plate obtained by Equation (3) is depicted in Figure 5. Because of the SEI formation, the modulus evolution in the 1st cycle is different from that in subsequent cycles. Here, we focus on analysis of the following cycles. As shown in Figure 5a. the modulus increases in the charging process, and reverses in the discharging process, which qualitatively agree with the observations reported before [28]. The modulus change here is an average value of the whole composite electrode, as a result of the comprehensive effect of the porosity and particle dilation. Due to accommodation of the dilation by porous structure, the modulus increases slowly in the early stage of charging. The further insertion of Li ions gives rise to a remarkable increase in modulus as attributed primarily to the modulus change of the material, thus leading to a decreasing slope as mentioned in Figure 4a.

Figure 5b shows the change of modulus along the electrochemical cycles with respect to different normalized concentration of 20%, 28%, 40% and 48%. At the same Li concentration, the electrode in the charging state has a larger modulus than that in the discharging state, which may imply that a degree of damage is caused by volume change after each cycle. It can also be concluded that the modulus declines gradually during multiple cycles, no matter in charging or discharging, which means composite graphite electrode going more cycles will result in severer degradation of mechanical property. Such degradation may be attributed to two aspects: one is cyclic volumetric expansion and contraction induced by insertion and extraction of Li ions within the active materials; the other is extreme bending deformation of electrode cantilever.

**Figure 6:**
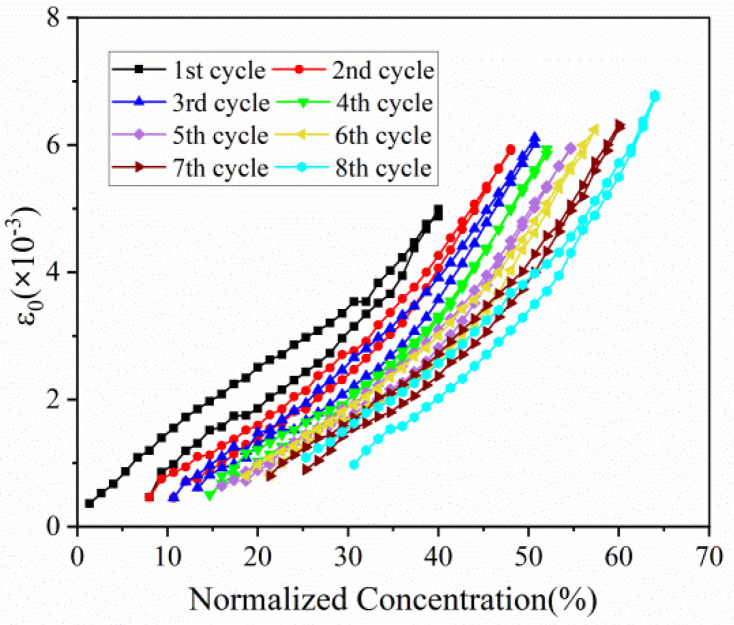
In-plain strain evolution of composite graphite electrodes corresponding to normalized concentration in 8 cycles.

Based on Equation (1), the evolution of in-plain strain with normalized capacity in the first eight cycles can be extracted, as displayed in Figure 6. The in-plain strain of composite electrode increases with the insertion of Li ions, and reverses in a similar path during discharging process. This phenomenon is in good accordance with stress evolution as follows. As for multiple cycles, strain variation trend is almost same and parallel. In addition, the maximum strain of each cycle, despite electrode in different concentration, remains at the almost same level, especially between the 2nd cycle and the 8th cycle.

**Figure 7:**
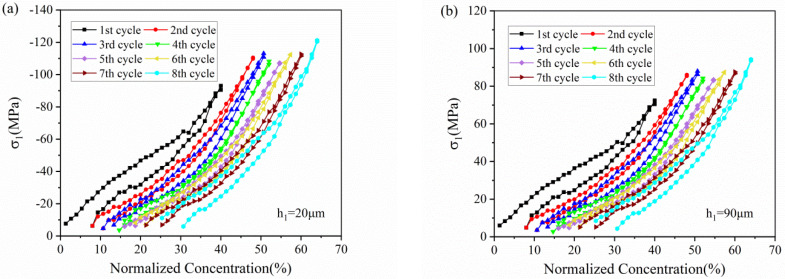
The evolution of stress in the active layer (**a**) in the thickness of 20 μm and (**b**) 90 μm corresponding to normalized concentration in 8 cycles.

**Figure 8:**
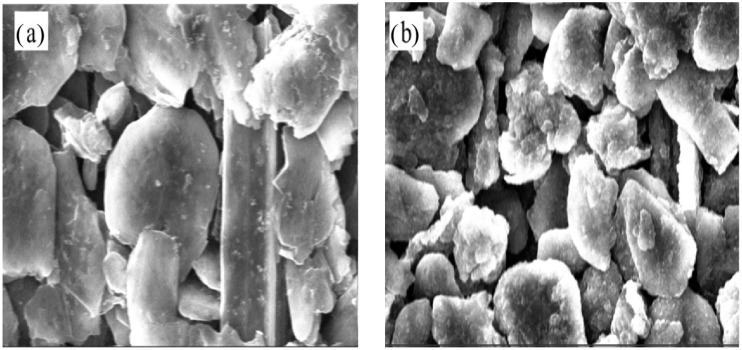
Microstructure of composite electrodes in the 2nd cycles corresponding to (**a**) SOC0% and (**b**) SOC40%.

Figure 7 depicts the stress variation of active layer along normalized concentration in thickness of 20 μm and 90 μm, respectively. With Li ions continuously migrating into the active material, the lithiated section tends to expand while the current collector and the unlithiated section tend to remain stationary. Hence, compressive stress is induced in the active layer near the interface (0 μm), where the expansion is constrained by the current collector, as shown in Figure 7a. However, bending-induced stress plays a more important role in the outer layer than diffusion induced stress, thus giving rise to tensile stress in the outer active layer, as shown in Figure 7b. Therefore, the stress of the active plate transforms from compression to tension along the thickness direction. The stress is not large enough to induce crack in the graphite particle, as demonstrated in Figure 8. However, it maybe affect the spatial structure of composite electrode.

All the stress in the active layer increase as the Li ion increasingly inserts into the active layer and decrease with the extraction of Li ions. It can also be found that the stress of each cycle exhibits same variation trend and varies within a limited range, no matter in compression or in tension. Such upper limit is ascribed to the structural damage in composite graphite electrode, contributing to the relief of stress induced by higher concentration.

**Figure 9:**
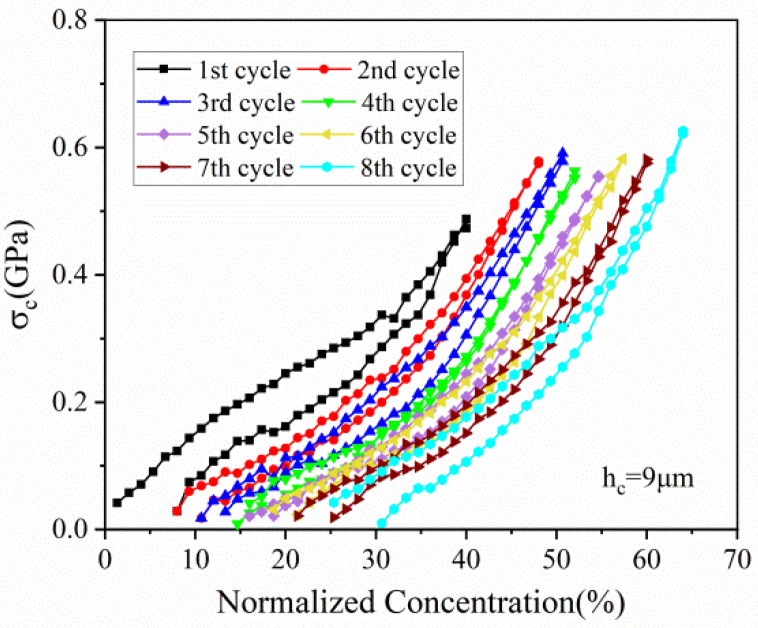
The evolution of stress in the current collector corresponding to normalized concentration in 8 cycles.

As demonstrated in Figure 9, tensile stress is generated in the current collector, which can be attributed to the traction of active plate. The stress of the current collector increases with the lithiation concentration, which is well consistent with the stress evolution of active layer. Moreover, the stress evolution of the current collector is parallel to each other cycle. Meanwhile, the magnitude of the tensile stress varies with concentration in a certain range, which shows the same phenomenon as the stress evolution in the active layer.

The hysteresis loops depicted in Figure 5, Figure 6, Figure 7, Figure 8 and Figure 9 may result from the damage induced by volume change during cycling. Therefore, the magnitude of the loop can provide a new avenue to reflect the damage state of the electrode. The first couple of cycles exhibit larger loops than the sequential, indicating that the composite graphite electrode generates severer damage at initial. The thresholds of stress/strain presented in Figure 6, Figure 7, Figure 8 and Figure 9 are the results of the co-interaction of concentration, damage and stress/strain. It could be a sort of comprehensive characterization of the property and damage of the electrode material.

## 4. Conclusions

The diffusion of Li ions, induced mechanical properties and stress are relevant to investigating the electro-chemo-mechanical coupled degradation and for further improving the cyclic performance of LIBs. Herein, in situ measurements of mechanical properties and stress evolution of commercial graphite composite electrodes are implemented during multiple cycles. The bending deformation of cantilever electrode is measured in real time, and the modulus and stress are quantified by the mechanical model developed in this study. The results have been discussed and analyzed from two perspectives.

From the single-cycle perspective, the evolutions of curvature, modulus, stress and strain are revealed in detail. The bending curvature increases almost linearly with the insertion of lithium ions in the charging process and undergoes two stages due to modulus evolution. A small residual curvature remains in the electrode after each cycle as attributed to SEI formation, residual Li-ions, microstructure change and electrode damage. The elastic modulus, stress and strain of the composite graphite electrode all increase nonlinearly with increasing lithiation, and decrease during delithiation with smaller values. Such processes present hysteresis loops associated with the damage state of the electrode. The stress of the active layer transforms gradually from compression to tension along the thickness direction.

From the multiple-cycle perspective, modulus and stress evolutions along the cycle number provide more insights to understand the electro-chemo-mechanical coupled degradation mechanism. The evolution of curvature, modulus, stress and strain during multiple cycles is parallel with each other, respectively, exhibiting the same trend, which indicates that composite graphite electrodes have good stability of structure, deformation and mechanical properties. Nevertheless, the internal stress induced by cyclic expansion and contraction and the external stress induced by bending deformation results in structural damage during cycling. This leads to the degradation of mechanical properties and the threshold of stress/stain. With the same Li concentration, the elastic modulus of composite graphite electrodes, in both the charging and discharging process, decreases continuously with the increase in the cycle number due to the accumulated structural damage. The magnitude of stress/strain varies in a limited range, and the maximum stress/strain of each cycle remains at the almost same level, reflecting a threshold of the stress/strain, respectively, at which the extra-stress induced by higher concentration is relieved due to damage. The threshold of the stress/strain is caused by the co-interaction of concentration, damage and stress/strain. Additionally, the magnitude of the threshold may depend on the electrode materials, geometrical dimensions and mechanical damage. The findings are conducive to better understanding the degradation mechanism of graphite electrodes as well as optimizing the design of LIBs.

## Data Availability

Experiment datas were under further consideration and will supplied later.
